# PyRhO: a virtual optogenetics laboratory

**DOI:** 10.1186/1471-2202-16-S1-P178

**Published:** 2015-12-18

**Authors:** Benjamin D Evans, Sarah Jarvis, Simon R Schultz, Konstantin Nikolic

**Affiliations:** 1 Institute of Biomedical Engineering, Department of Electrical & Electronic Engineering, Imperial College London, London, UK; 2 Department of Bioengineering, Electrical Engineering, Imperial College London, London, UK

## 

Optogenetics has become a key tool for understanding the function of neural circuits and controlling their behaviour. An array of opsins have been genetically isolated from several families of organism, including algae and bacteria, with a wide range of temporal and spectral properties. In an effort to develop more effective and tailored opsins, hybrids and genetic mutants are continually being created. Experimentally characterizing these new variants is a lengthy process requiring substantial effort before they can be harnessed to address questions in neuroscience. Experimentally testing each combination of opsin and target cell type of interest is practically impossible, effectively limiting the use of optogenetics as a tool. To aid in this effort we propose *PyRhO*; an integrated suite of open-source, multi-scale computational tools to characterize rhodopsins, then rapidly develop and conduct virtual experiments with them *in silico*.

From a minimal set of photocurrent data, *PyRhO *will fit and parameterize the Three [1], Four [1] and Six-state [2] rhodopsin models to capture the underlying biophysical photocycle which defines their kinetics. These models are then used to accurately compute the photocurrents across a range of flux, voltage and other experimental conditions for the given rhodopsin. After selecting a suitable model based on the desired balance between simulation accuracy and speed, the artificial rhodopsin can be seamlessly inserted into software such as NEURON and Brian for use in simulations from the cellular to the network level. We demonstrate the use of *PyRhO *in fitting models to channelrhodopsin-2 (ChR2) [3] data and present results for typical illumination strategies and experimental protocols designed to tease apart the effects of key model parameters.

The tools are written in Python for easy scripting of experiments and compatibility with a large array of open-source modules and software. An accompanying GUI running in IPython [4] has also been developed to facilitate more interactive exploration of the models for both experimental and didactic purposes. Furthermore, IPython has been identified as a particularly promising medium for sharing models and reproducing results in computational neuroscience [5]. Simulations based on these virtual opsins will enable neuroscientists to gain insight into their behaviour and rapidly identify the most suitable variant for application in a particular biological system, not only guiding choice, but potentially also rhodopsin development. In this way, we expect *PyRhO *will help to significantly improve the effectiveness of optogenetics as a tool for transforming biological sciences.
